# Wall motion in the stenotic carotid artery: association with greyscale plaque characteristics, the degree of stenosis and cerebrovascular symptoms

**DOI:** 10.1186/1476-7120-11-37

**Published:** 2013-10-20

**Authors:** Baris Kanber, Timothy C Hartshorne, Mark A Horsfield, Andrew R Naylor, Thompson G Robinson, Kumar V Ramnarine

**Affiliations:** 1 Department of Cardiovascular Sciences, University of Leicester, Leicester, UK; 2 Department of Surgery, University Hospitals of Leicester NHS Trust, Leicester, UK; 3 NIHR Biomedical Research Unit for Cardiovascular Sciences, University of Leicester, Leicester, UK; 4 Department of Medical Physics, University Hospitals of Leicester NHS Trust, Leicester, LE1 5WW UK

**Keywords:** Ultrasound, Carotid artery, Carotid plaque, Stenosis, Wall motion, Distension, Dilation, Greyscale median, GSM, Surface irregularity index, SII, Cerebrovascular symptoms

## Abstract

**Background:**

Systolic dilation of the atherosclerotic carotid artery depends on several factors including arterial compliance and the haemodynamic environment. The purpose of this study was to quantify wall motion in stenotic carotid arteries and investigate any associations with the ultrasound greyscale plaque characteristics, the degree of stenosis, and the presence of cerebrovascular symptoms.

**Methods:**

Variations in the lumen diameters of 61 stenotic carotid arteries (stenosis range 10%-95%) from 47 patients were measured before the proximal shoulder of the atherosclerotic plaque using ultrasound image sequences over several cardiac cycles. Absolute and percentage diameter changes from diastole to systole were calculated and their relationship to the degree of stenosis, greyscale plaque characteristics, and the presence of ipsilateral hemispheric symptoms were studied.

**Results:**

The mean absolute diameter change from diastole to systole was 0.45 mm (s.d. 0.17), and the mean percentage diameter change was 6.9% (s.d. 3.1%). Absolute and percentage diameter changes did not have a statistically significant relationship to the degree of stenosis, greyscale plaque characteristics, or the presence of ipsilateral hemispheric symptoms (p > 0.05). Parameters significantly correlated with the presence of symptoms were the degree of stenosis (p = 0.01), plaque greyscale median (p = 0.02) and the plaque surface irregularity index (p = 0.02).

**Conclusions:**

Our study confirmed the degree of stenosis, plaque greyscale median and our surface irregularity index were significant predictors of symptoms, but found no significant correlation between diameter changes of stenosed carotid arteries and the presence of ipsilateral hemispheric symptoms.

## Introduction

Patients attending transient ischaemic attack (TIA) clinics often undergo ultrasound imaging of their carotid arteries, during which the presence of any atherosclerotic plaques are noted and the corresponding degrees of stenosis are measured. The degree of stenosis quantifies the severity of the stenosis and is used routinely in clinical practice. However, there is growing demand for additional parameters which can further differentiate high-risk or vulnerable plaques, particularly in those with low to moderate degrees of stenosis. Studies have found that plaque composition in patients undergoing carotid endarterectomy is an independent predictor of future cardiovascular events with plaque neovascularisation and haemorrhage relating to adverse cardiovascular outcome during follow-up [1]. Greyscale plaque characteristics, such as the plaque greyscale median (GSM) and surface irregularities also have the potential to be additional indicators of vulnerable plaques [2-10]. Ultrasound assessment of the stiffness of the carotid plaque using shearwave elastography is an emerging technique that may also provide additional benefit [11]. Another potentially useful parameter which can be easily measured from ultrasound scans performed in the TIA clinic is the systolic dilation or distension of the artery with the atherosclerotic plaque. The dilation of the carotid artery from diastole to systole depends on several factors including arterial stiffness, and previous studies have shown that stiffer arteries are associated with atherosclerosis and are risk factors for stroke and other cardiovascular diseases [12-15]. The amount of arterial dilation is a physical parameter that may also affect the stability of the plaque, since greater arterial motion may increase the mechanical stress on the plaque and promote instability [16-20].

The presence of the atherosclerotic plaque can have a significant effect on arterial wall motion [21,22]. One study found that arterial distensibility was not only significantly lower in the internal carotid artery where there was a plaque, but it was also lower in the common carotid artery of the affected side in comparison with the contralateral common carotid artery, providing evidence that the effect of a plaque on arterial mechanical properties is not limited to the actual plaque site but rather extends to a considerable degree in a proximal direction [21]. Computational models, on the other hand, showed that the non-uniform thickness of the diseased arterial wall can restrict wall motion and re-distribute stress, giving rise to increased stress concentrations at the plaque shoulders [22]. Therefore, an assessment of the wall motion characteristics of the stenosed carotid artery may provide useful indicators that may correlate with the risk of plaque rupture and the prevalence of symptoms.

A previous study found that patients with acute symptomatic carotid stenosis had impaired brachial flow mediated dilation (FMD) compared to patients with asymptomatic carotid stenosis in a patient population with greater than 50% reduction in the diameter of the carotid artery [23]. That study showed that impaired brachial FMD was an independent predictor of cerebral ischaemic symptoms. However, not many studies have considered whether the physiological dilation of the stenosed carotid artery itself might have any correlation to cerebrovascular symptoms, addressing the question of whether patients presenting with ipsilateral hemispheric symptoms have distinctly different carotid artery dilations compared to patients that do not. A study by Ramnarine et al. [24] looked at the physiological dilation of atherosclerotic carotid arteries and correlated results with the degree of stenosis, but any relationships to the presence of patient symptoms were not investigated. Another study examined the dilation characteristics of the carotid artery at the level of the plaque and compared this with the adjacent common carotid artery leading to a longitudinal strain gradient estimation, but again, any relationships to the presence of patient symptoms were not studied [25]. More recently, Beaussier et al. [26] studied the longitudinal distension gradient between the plaque and the adjacent common carotid artery with respect to the presence of ipsilateral hemispheric symptoms and found no statistically significant differences. However, their results do not appear to indicate whether there were any significant differences in degree of arterial dilation at the adjacent carotid segment between the symptomatic and asymptomatic groups. That particular study also involved only a small number of carotid arteries with ipsilateral symptoms (n = 9).

It is plausible that wall motion in the stenotic carotid artery may affect the stability of the carotid plaque and consequently, relate to the presence of ipsilateral hemispheric symptoms. Wall motion data along with the degree of stenosis and greyscale plaque characteristics may help identify the vulnerable plaque. The purpose of this study was, therefore, to test the hypothesis that the systolic dilation of the stenosed carotid artery is related to the presence of ipsilateral hemispheric symptoms and can be used to differentiate between symptomatic and asymptomatic patients. Arterial wall motion was measured before the proximal shoulder of the plaque as this is an ideal, upstream location close to the plaque where a well defined segment of the artery can often be found. The latter is important because arterial wall motion measurements across the plaque can suffer from high variability [24]. Our investigation measured the absolute and percentage dilation of stenotic carotid arteries, from end diastole to peak systole, and explored whether these parameters had any statistically significant associations to the degree of stenosis, greyscale plaque characteristics, and the presence of ipsilateral hemispheric symptoms.

## Methods

Forty seven patients who attended the University Hospitals of Leicester NHS Trust’s Rapid Access Transient Ischaemic Attack clinic were recruited. Variations in the lumen diameters of 61 stenotic carotid arteries (stenosis range 10%-95%) were measured. The study was approved by the National Research Ethics Service (NRES) Committee East Midlands - Northampton (reference 11/EM/0249) and followed institutional guidelines. Each patient gave informed consent before participating in the study. Patients who did not have carotid artery stenosis were excluded from the study. Carotid arteries for which the ultrasound image quality was poor were excluded from the wall motion analysis. Image sequences which were considered to be of poor quality included those with substantial image noise in the vessel lumen and those with poorly defined vessel wall segments. In the case of patients with stenosed left/right carotid arteries, each side was included and analyzed separately. In total, lumen diameter variations of 45 stenosed carotid arteries were included in the final wall motion analysis. Carotid arteries with atherosclerotic plaque were classified as either having ipsilateral hemispheric cerebrovascular symptoms (i.e. symptomatic) or asymptomatic following specialist medical review. Symptoms included aphasia, transient monocular blindness and hemimotor/sensory symptoms consistent with transient ischaemic attack or stroke.

### Data acquisition

Longitudinal cross-sections of the carotid artery and plaque were imaged by experienced sonographers using a Philips iU22 ultrasound scanner (Philips Healthcare, Eindhoven, The Netherlands) and an L9-3 probe. Acquisitions included B-Mode (i.e. greyscale) and Colour Doppler image sequences. B-Mode image sequences were acquired using the vascular carotid preset on the scanner (Vasc Car preset, persistence low, XRES and SONOCT on) and were recorded in DICOM format over an average of 6 cardiac cycles (mean frame rate was 32 frames per second). Gain was optimized by the experienced sonographers. In the case of B-Mode acquisitions, the greyscale transfer curve was set to Gray Map 2, as this was reported to be the most linear transfer curve on this scanner [27]. Colour Doppler cine-loops were used as a qualitative aid to identifying the location and extent of carotid plaques, while the B-Mode data were used for the quantitative analyses including that of arterial wall motion and greyscale plaque characteristics.

### Analysis

Quantitative analyses were carried out using MATLAB version 7.14, release 2012a (MathWorks, Natick, Massachusetts, USA) and employed an image processing algorithm to track and measure arterial lumen diameters over time. This algorithm was based on a probabilistic approach to vessel lumen segmentation [28]. In this approach, given a point B with probability P_b_ of being in the arterial lumen of interest, the probability P_a_ that a neighbouring point A was also part of the same lumen was proportional to P_b_ with a Gaussian fall in probability with increasing greyscale contrast between the two points (Equation 1). Here G_b_ and G_a_ were the greyscale intensities of points B and A, and the constant ζ was determined by considering the amount of greyscale contrast (G_th_) required to reduce P_a_ to 1/2 that of P_b_.

(1) $$P_{a}=P_{b}\;\exp\;\left(-{\left(G_{b}-G_{a}\right)}^{2}/\zeta \right)\;\mathrm{where}\;\zeta =G_{\mathrm{th}}{}^{2}/\log\;2$$

The arterial lumen detection technique based on this algorithm was previously found to have good arterial wall tracking performance, comparable to that of Tissue Doppler Imaging [29].

**Figure 1:**
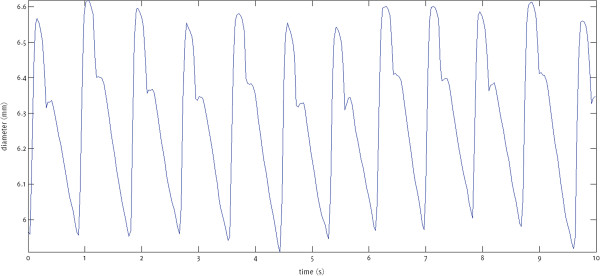
Example of an arterial dilation waveform showing lumen diameter variations of a carotid artery throughout several cardiac cycles.

**Figure 2:**
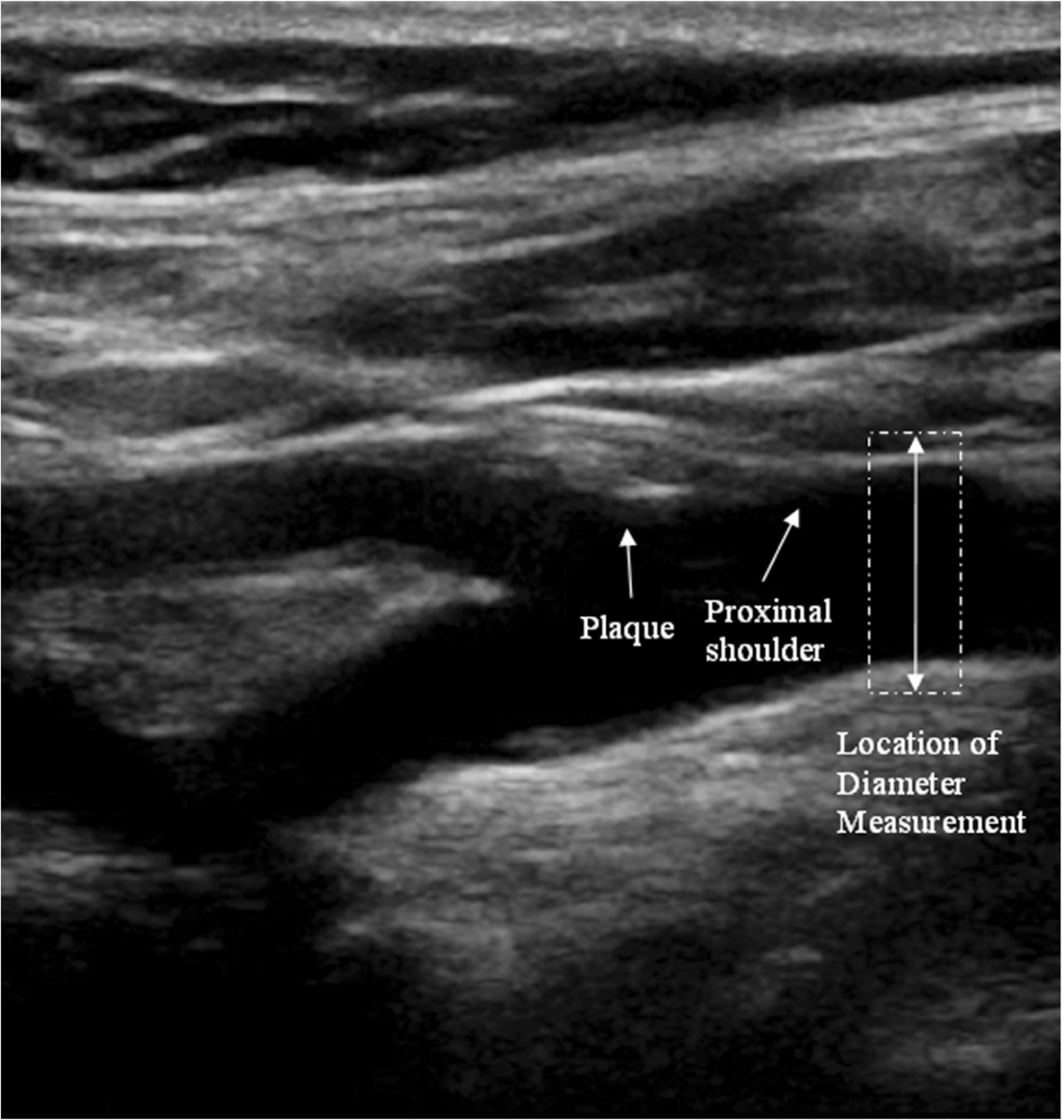
**A carotid bifurcation plaque and illustration of the location of the diameter measurements.** In this case, the plaque appears on the carotid bulb, and diameter measurements are taken in the distal common carotid artery immediately before the proximal shoulder of the plaque.

Arterial diameter variation waveforms (Figure 1) were obtained before the proximal shoulder of the plaque, but as close to it as possible, and averaged over a region approximately 3 mm long for each image frame (Figure 2). The measurements were made without prior knowledge of the patient’s symptomatic status. The peaks of the diameter variation waveforms (Figure 1) were taken to be the (peak) systolic values and the troughs as the (end) diastolic. The absolute value of the systolic arterial dilation was calculated as the increase in the arterial lumen diameter from diastole to systole and percentage systolic dilation as the same figure divided by the diastolic diameter. The same calculations were carried out for all the cardiac cycles observed on the arterial diameter variation waveforms and averages were taken. Normalized and un-normalized plaque GSM and surface irregularity indices (SII) were obtained using previously described methods [8,10] while the degree of stenosis of the corresponding arteries were measured using criteria consistent with the NASCET method utilizing blood flow velocities in conjunction with the B-Mode and colour flow imaging [30-32].

### Statistical analysis

Statistical analyses were carried out using SPSS version 20 (IBM Corporation, Armonk, New York, USA). The non-parametric Wilcoxon-Mann–Whitney test was used to determine whether quantitative measurements such as the absolute and percentage diameter changes, degree of stenosis, and greyscale plaque characteristics differed significantly between patient groups (e.g. symptomatic/asymptomatic, hypertensive/normotensive, etc.). A further ANCOVA test was carried out between the percentage systolic dilation of the artery and symptomatic status, controlling for the effects of the diastolic arterial diameter. Pearson’s correlation was used to determine whether the absolute and percentage diameter changes had any statistical relationship to the age of the patient, the degree of stenosis, and the greyscale plaque characteristics. Partial correlations, controlling for the effects of the baseline diastolic diameters, were also carried out for the percentage diameter changes. Finally, logistic regression was carried out to investigate which parameters significantly correlated with the presence of ipsilateral hemispheric symptoms. Two-tailed values of significance were used and values less than 0.05 were considered to be statistically significant.

### Reproducibility

In order to assess the reproducibility of the arterial wall motion detection technique used, we investigated the intra-observer coefficients of variation for the measurement of the systolic/diastolic diameters, and absolute/percentage diameter changes for 10 arteries. This subset of arteries were selected from the available dataset to give a wide range of stenosis severity, plaque echogenicity and arterial diameters for reproducibility analysis. The measurements were made by the same operator and in sequential order. The same ultrasound acquisition sequences were used for each artery respectively.

### Comparison against manual measurements

Arterial diameter measurements made using our method were compared against diameter measurements made manually by placing cursors on the ultrasound images and measuring the distances between the near and far walls of the arteries. This was done using a computer program with a graphical user interface written in MATLAB version 7.14, release 2012a (MathWorks, Natick, Massachusetts, USA). Using the same arteries selected for our reproducibility analysis, we manually measured arterial diameters at the same location and on matching image frames as for the automated technique. Approximately 30 diameter measurements per artery spread over the cardiac cycles were compared between the two techniques. Arterial diameters obtained using the manual method were compared with those obtained using the automated technique using Bland-Altman and linear regression analysis.

## Results

Mean age was 77.3 years (range 58–95 years); 19 female. The prevalence of cerebrovascular risk factors was: 76% hypertension, 53% hypercholesterolaemia, 33% ischaemic heart disease, 29% diabetes mellitus, 47% previous TIA/stroke, 64% smoking, 44% alcohol consumption and 33% family history of stroke. Thirty one of the 61 arteries studied were found to be associated with ipsilateral hemispheric symptoms, while the remaining 30 were found to be asymptomatic following expert specialist stroke physician assessment.

**Figure 3:**
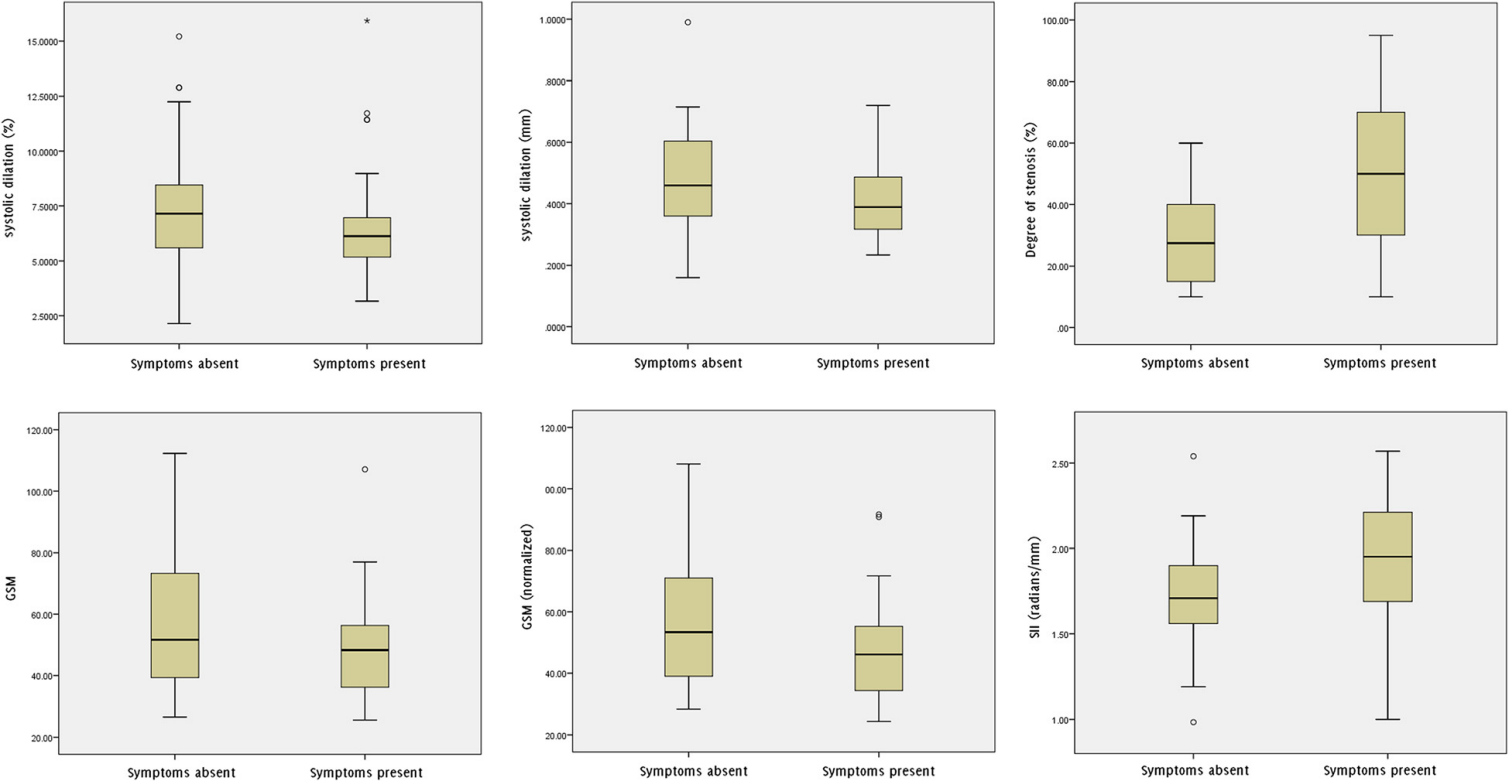
Box and whisker plots showing the distribution, versus the presence of ipsilateral hemispheric symptoms, of the absolute and percentage arterial diameter changes, degree of stenosis, normalized and un-normalized plaque GSM, and the surface irregularity index (SII).

The mean percentage systolic dilation of the symptomatic arteries (6.6%) was lower than that of the asymptomatic arteries (7.2%), but this difference was not statistically significant (p = 0.16, Figure 3). ANCOVA, controlling for the effects of the diastolic diameters, also found the same difference to be not statistically significant (p = 0.14). Arteries with ipsilateral hemispheric symptoms also had lower absolute diameter changes on average (0.42 mm) than asymptomatic arteries (0.47 mm) but this difference was also not significant (p = 0.17, Figure 3). The degree of stenosis (p < 0.01), normalized plaque GSM (p = 0.021) and the plaque surface irregularity index (p = 0.016) differed significantly between the symptomatic and asymptomatic groups while the un-normalized plaque GSM (p = 0.14) did not.

**Figure 4:**
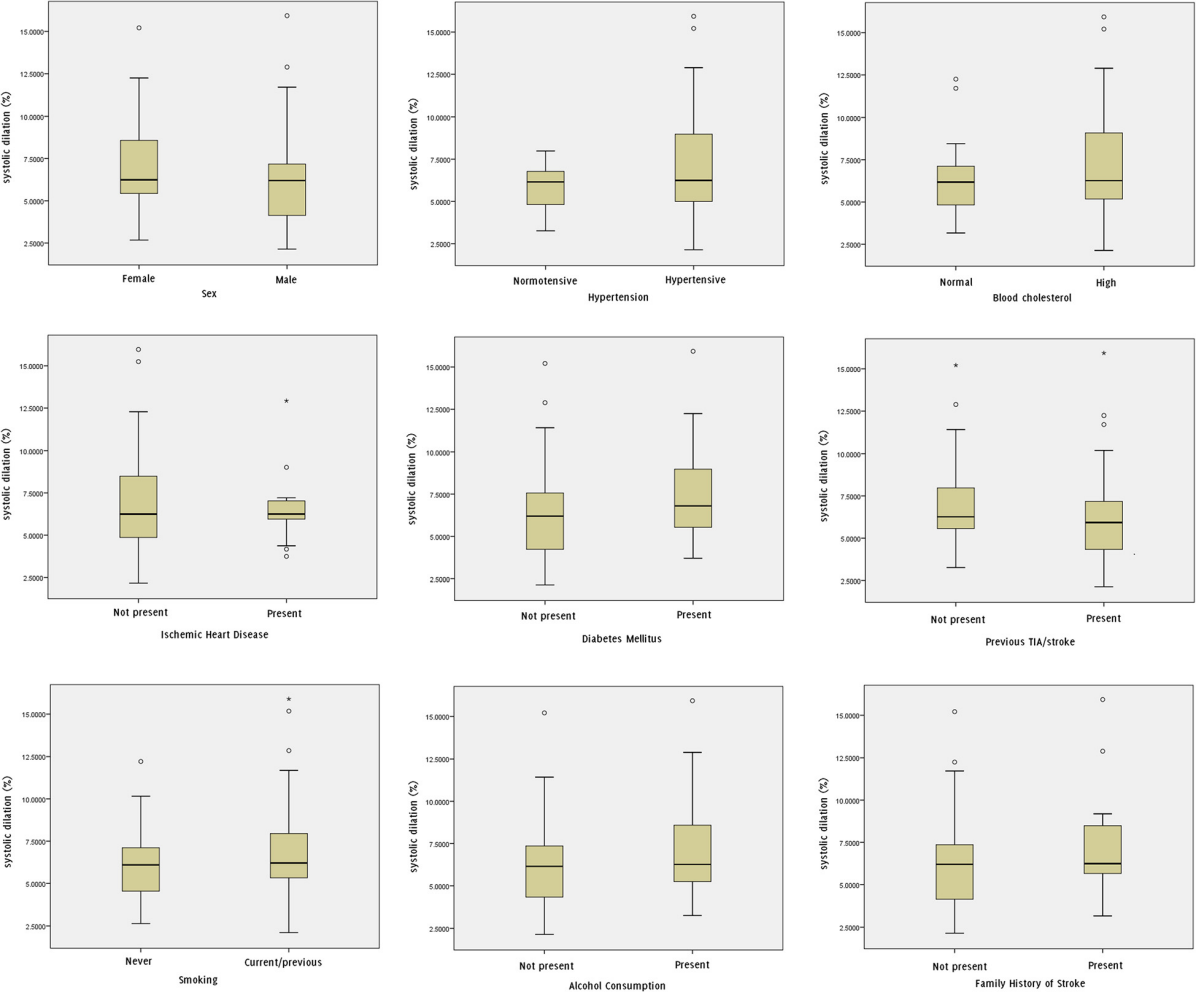
Box and whisker plots showing the distribution of the percentage systolic diameter changes versus patient characteristics.

**Figure 5:**
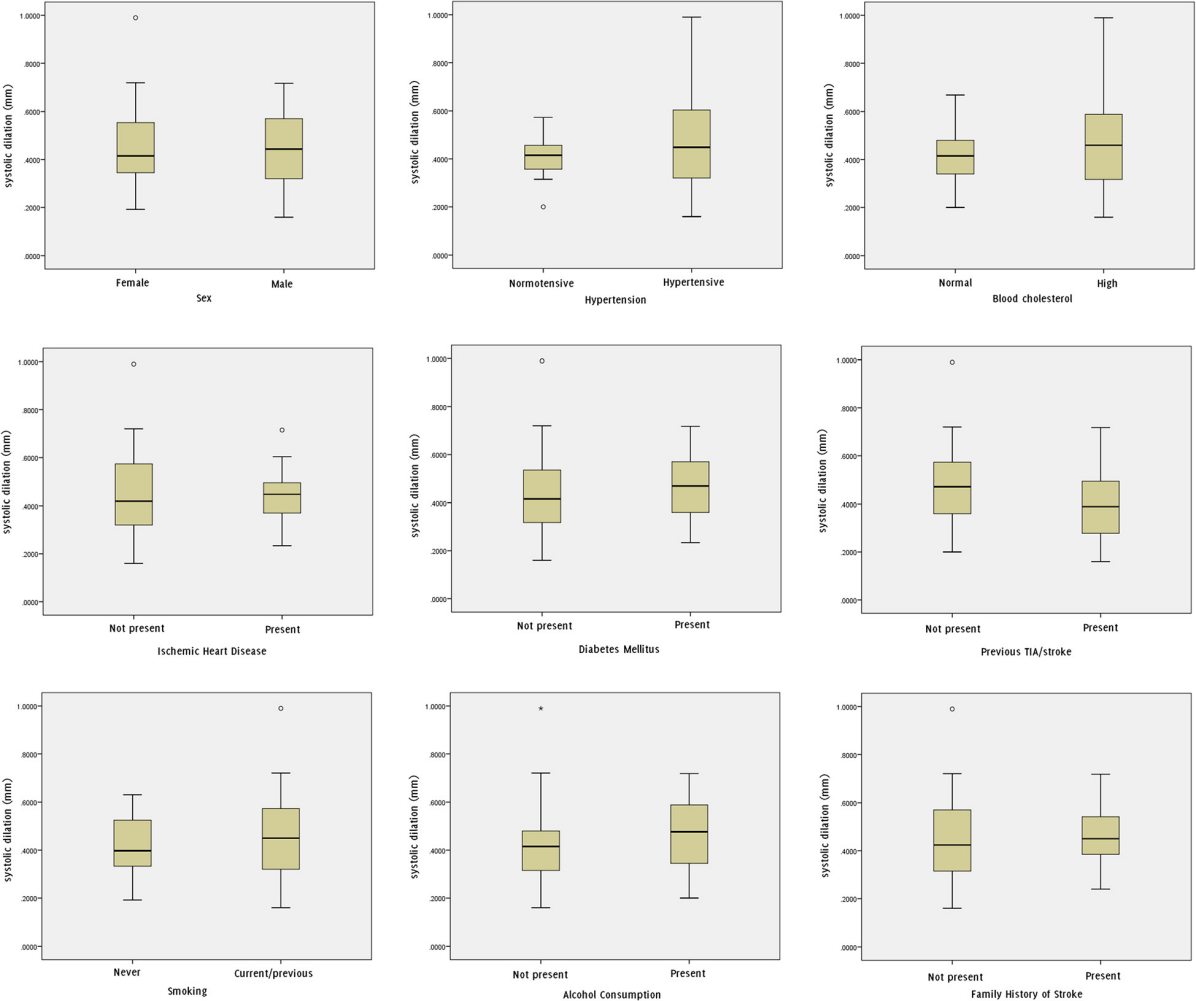
Box and whiskers plots showing the distribution of the absolute systolic diameter changes versus patient characteristics.

**Figure 6:**
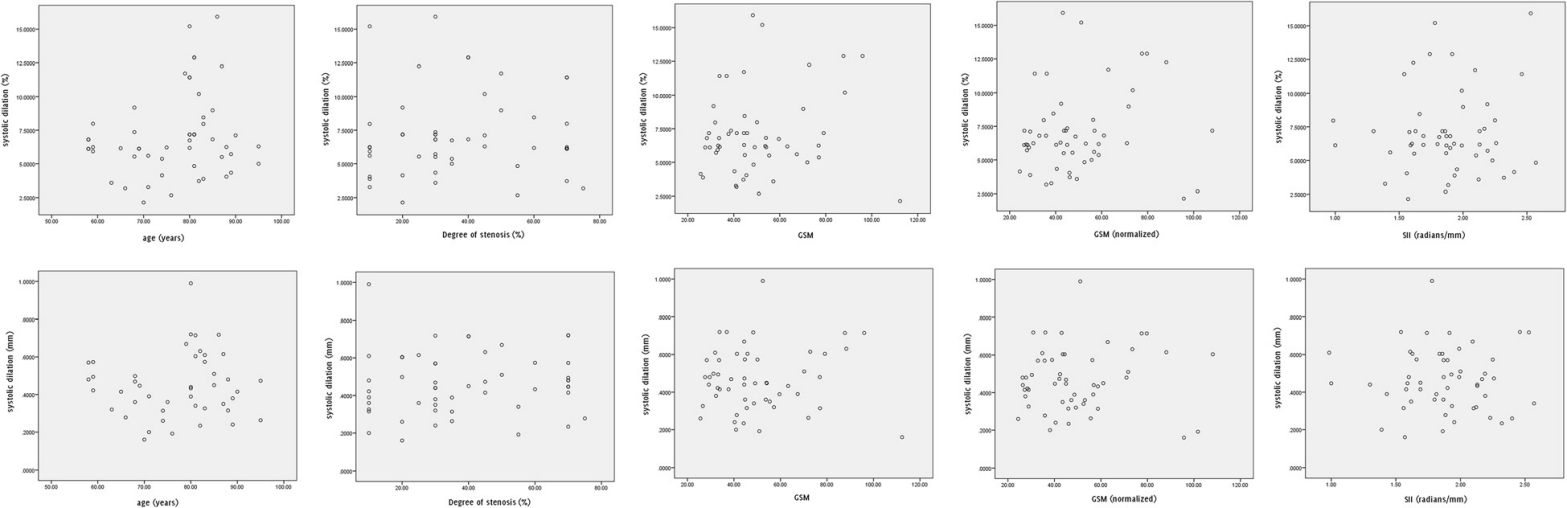
Scatter plots of the absolute and percentage systolic diameter changes versus patient age, degree of stenosis, un-normalized and normalized plaque GSM, and the plaque surface irregularity index (SII), illustrating a lack of association between the absolute and percentage systolic dilation and any of these parameters.

Patient characteristics age, sex, hypertension, high cholesterol, ischaemic heart disease, diabetes mellitus, previous TIA/stroke, smoking, alcohol consumption, and family history of stroke did not show significant differences in the percentage and absolute systolic dilation of the arteries (Table 1, Figures 4 and 5). There were no statistically significant correlations between the percentage systolic dilation of arteries and the degree of stenosis (p = 0.82), patient age (p = 0.14), un-normalized plaque GSM (p = 0.29), normalized plaque GSM (p = 0.34) or the plaque surface irregularity index (p = 0.54, Figure 6). Partial correlations, adjusting for the effects of the baseline diastolic diameters, also found no statistically significant relationship between the percentage systolic dilation of the arteries and the degree of stenosis, patient age, or the greyscale plaque characteristics (p > 0.05 for all). Absolute diameter changes were also not significantly correlated with the degrees of stenosis (p = 0.70), patient age (p = 0.68), un-normalized plaque GSM (p = 0.78), normalized plaque GSM (p = 0.69) or the plaque surface irregularity index (p = 0.90, Figure 6).

**Table 1:** Non-parametric Wilcoxon-Mann–Whitney associations between the absolute and percentage systolic diameter changes before the proximal shoulder of the atherosclerotic plaque and patient characteristics

**Patient characteristic**	**Significance (p-value)**	
	**Absolute diameter change**	**Percentage diameter change**
Age	0.44	0.33
Sex	0.96	0.68
Hypertension	0.45	0.34
Hypercholesterolaemia	0.54	0.41
Ischaemic heart disease	0.94	0.90
Diabetes mellitus	0.31	0.28
Previous TIA/stroke	0.16	0.32
Smoking	0.34	0.49
Alcohol	0.29	0.47
Family history of stroke	0.35	0.41

Age was dichotomized using the median of the dataset as a cut-off value.

Logistic regression testing found only the degree of stenosis, normalized plaque GSM and the surface irregularity index to be significant predictors of the presence of ipsilateral hemispheric symptoms (Table 2). The un-normalized plaque GSM was not found to have a significant correlation to symptoms.

**Table 2:** Logistic regression testing for any association between the presence of ipsilateral hemispheric symptoms and the degree of stenosis, greyscale plaque characteristics and the absolute and percentage dilation of the arteries

**Parameter**	**Significance (p-value)**
Degree of stenosis	0.01*
Percentage systolic diameter change	0.20
Absolute systolic diameter change	0.10
GSM	0.09
GSM (normalized)	0.02*
SII	0.02*

Significant associations are marked with an asterisk (*).

**Figure 7:**
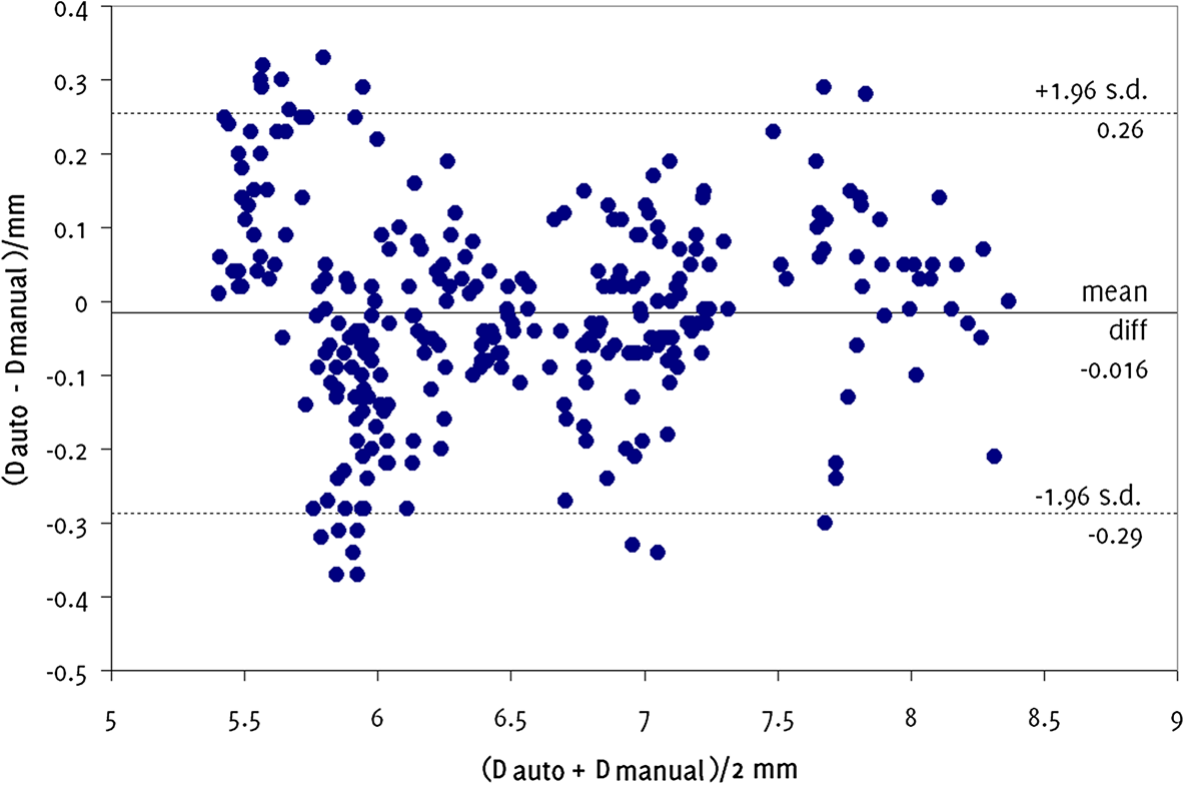
**Bland-Altman plot showing the differences in arterial diameters, on matching image frames, measured manually (D**_**manual**_**) and using our method (D**_**auto**_**).**

**Figure 8:**
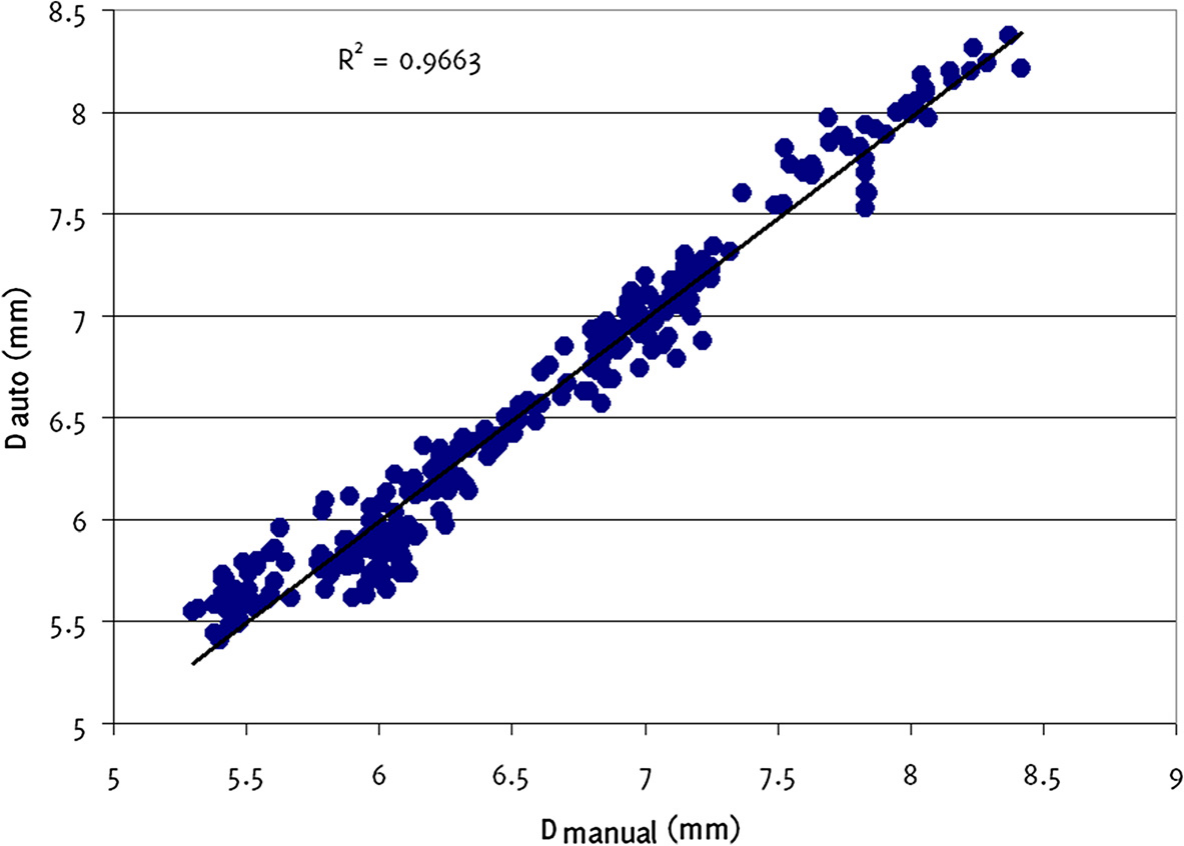
**Scatter plot showing a strong linear relationship between arterial diameters measured manually (D**_**manual**_**) and using our method (D**_**auto**_**).**

Our assessment of reproducibility showed mean intra-observer coefficients of variation of 1.0%, 1.2%, 11.7%, and 12.4% for the measurement of the systolic diameters, diastolic diameters, absolute systolic diameter changes, and percentage diameter changes, respectively. Comparison against manual measurements showed a mean difference in diameter measurements between the two techniques of −0.016 mm (Figure 7) which did not differ significantly from zero (p = 0.06, t-test). The 95% limits of agreement were −0.29 mm to 0.26 mm. Linear regression analysis showed a strong correlation between the measurements made using the two methods (R^2^ = 0.97, Figure 8).

## Discussion

This study provides new data on wall motion in atherosclerotic carotid arteries and the association with cerebrovascular symptoms, the degree of stenosis, and greyscale plaque characteristics. These data may also be useful for informing computational models of carotid stenosis [33] and experimental phantom replicas [34,35], especially considering the scarcity of distension measurements immediately before the proximal shoulder of the carotid plaque.

A motivation for our study was the plausibility of a relationship between the diastolic to systolic dilation of the carotid artery and the presence of cerebrovascular symptoms, since changes in arterial wall motion behaviour may be indicative of vascular disease and aging, both of which are risk factors for stroke [12,13,36]. However, our study found no significant relationship between the absolute and percentage dilation of the carotid artery before the proximal shoulder of the atherosclerotic plaque and the presence of cerebrovascular symptoms. Diameter changes were also not significantly correlated with the degree of stenosis, in accordance with our previous findings [24].

Arteries which have greater amounts of wall motion may increase plaque vulnerability due to mechanical factors [16-18]. Therefore, with progressive atherosclerotic disease, while the risk of stroke may be raised on a systemic level, a reduction in the amount of arterial wall motion may result in a lower risk of plaque rupture from a mechanical perspective. These considerations complicate the relationship between the dilation characteristics of the carotid artery before the proximal shoulder of the atherosclerotic plaque and the presence of cerebrovascular symptoms, and are likely to be factors that contribute to the absence of a difference in the carotid artery dilations of the symptomatic and asymptomatic patients found in this study.

Arterial lumen diameters measured using our technique were found to be comparable to those measured using a manual method. Our study found good reproducibility for the measurement of the diastolic and systolic diameters but lower reproducibility for the measurement of the absolute and percentage diameter changes. These results are in accordance with previous studies which found derived parameters combining the systolic and diastolic arterial diameters to be considerably less reproducible than to the diameter readings on their own [37,38]. It has been reported that even a small variance in arterial diameter measurements may cause a considerable variance in the derived metrics of carotid distension, therefore, limiting its potential usability in the clinical setting [38]. Godia et al. attributed the different and sometimes conflicting results reported on the association between carotid distension and cardiovascular outcomes to this variability [38]. In the present study, the greater variabilities associated with absolute and percentage diameter changes may be additional factors contributing to the absence of a difference found in the carotid artery dilations of symptomatic and asymptomatic patients. Studies incorporating larger datasets or more precise methods may be able to find such a difference.

The statistically significant relationship between the presence of ipsilateral hemispheric symptoms and both the normalized plaque GSM and the surface irregularity index confirm our previous findings [8,10]. Interestingly, the un-normalized plaque GSM was not found to be a significant predictor of symptoms. This may be indicative of variations in overall image brightness, due to differences in ultrasound gain settings or tissue attenuation, and highlights the importance of the normalization procedure for GSM measurements.

A limitation of this study is that we did not have pulse pressure measurements which would have allowed us to quantify arterial distensibility. However, this study focussed on motion aspects rather than stiffness and is part of our broader research aim to develop and define a plaque risk index based on ultrasound measurements. Previously, we have quantified greyscale plaque characteristics such as the plaque GSM and surface irregularities [8,10] as possible indicators of vulnerable plaques and the present study was conducted to investigate the potential of dilation characteristics before the proximal plaque shoulder as an additional parameter to include in a prospective vulnerable plaque-stroke risk model. In this study we did not perform measurements across the plaque to assess any differential wall motion between the plaque and the proximal carotid segment. Our previous study using Tissue Doppler Imaging demonstrated a variety of pertinent wall motion features across the plaque site that may be related to the biophysics of arterial disease. However, high variability demonstrated the limitations of arterial wall motion measurements across the plaque, in contrast to more robust measurements that can be performed on well defined segments of vessels [24].

## Conclusions

This study investigated the systolic dilation of stenosed carotid arteries measured before the proximal shoulder of the atherosclerotic plaque. Absolute and percentage diameter changes were lower for the arteries of patients with ipsilateral hemispheric symptoms, but these differences were not statistically significant. Normalized plaque GSM and our novel surface irregularity index were found to be significant predictors of symptoms.

## Competing interests

The authors declare that they have no competing interests.

## Authors’ contributions

The study was conceived by KVR and BK. Ultrasound data were collected by TCH while the analyses were carried out by BK. All authors contributed to the interpretation and presentation of the results and, read and approved the final manuscript.
